# Comparative Transcriptome Analysis of the Heterosis of Salt Tolerance in Inter-Subspecific Hybrid Rice

**DOI:** 10.3390/ijms24032212

**Published:** 2023-01-22

**Authors:** Zhibo Huang, Jing Ye, Rongrong Zhai, Mingming Wu, Faming Yu, Guofu Zhu, Zhoufei Wang, Xiaoming Zhang, Shenghai Ye

**Affiliations:** 1 Institute of Crop and Nuclear Technology Utilization, Zhejiang Academy of Agricultural Sciences, Hangzhou 310021, China; 2 The Laboratory of Seed Science and Technology, Guangdong Key Laboratory of Plant Molecular Breeding, College of Agriculture, South China Agricultural University, Guangzhou 510642, China

**Keywords:** *Oryza sativa*, inter-subspecific hybrid, salt tolerance, heterosis

## Abstract

Soil salinity is one of the major abiotic stresses limiting rice growth. Hybrids outperform their parents in salt tolerance in rice, while its mechanism is not completely understood. In this study, a higher seedling survival was observed after salt treatment in an inter-subspecific hybrid rice, Zhegengyou1578 (ZGY1578), compared with its maternal *japonica* Zhegeng7A (ZG7A) and paternal *indica* Zhehui1578 (ZH1578). A total of 2584 and 3061 differentially expressed genes (DEGs) with at least twofold changes were identified between ZGY1578 and ZG7A and between ZGY1578 and ZH1578, respectively, in roots under salt stress using the RNA sequencing (RNA-Seq) approach. The expressions of a larger number of DEGs in hybrid were lower or higher than those of both parents. The DEGs associated with transcription factors, hormones, and reactive oxygen species (ROS)–related genes might be involved in the heterosis of salt tolerance. The expressions of the majority of transcription factors and ethylene-, auxin-, and gibberellin-related genes, as well as peroxidase genes, were significantly higher in the hybrid ZGY1578 compared with those of both parents. The identified genes provide valuable clues to elucidate the heterosis of salt tolerance in inter-subspecific hybrid rice.

## 1. Introduction

Rice is one of the most important cereal grains in China. Hybrid rice occupies more than 50% of the total rice area due to its heterosis advantage [1]. Heterosis or hybrid vigor refers to the phenomenon in which hybrids outperform their parents in yield, biomass, and stress tolerance. Previous studies were mainly focused on the root heterosis, grain number heterosis, and grain weight heterosis in *indica* intra-subspecific hybrid rice [2,3,4]. Inter-subspecific *indica* × *japonica* hybrid rice shows a significant degree of heterosis [5], but very few studies have been conducted on the heterosis of salt tolerance. Soil salinity is one of the major abiotic stresses limiting rice growth and yield [6]. Therefore, the breeding of salt-tolerant *indica* × *japonica* hybrid is one of the most economic options to ensure food security [7]. 

The genetic mechanisms of heterosis have been extensively studied in crops. Three classical genetic hypotheses, including dominance [8], overdominance [9], and epistasis [10,11], have been considered as explanations for the genetic basis of heterosis. The dominance hypothesis states that the undesirable recessive alleles from one parent can be complemented by the dominant beneficial alleles from another parent [8,12]. The overdominance hypothesis means that the intra-allele interaction explaining the fitness of individuals carrying heterozygous genotypes is higher than those carrying a homozygous genotype [9,13]. The epistasis hypothesis indicates that the inter-allele interaction among multiple genes and pathways is the reason for heterosis [10,11]. In rice, genome-wide association studies have been performed to explain the underlying mechanism of dominance and overdominance effects relevant to heterosis [14]. The inter-subspecific hybrids obviously have higher heterosis than that of the intra-subspecific hybrids [15]. However, the underpinning heterosis mechanism in the inter-subspecific *indica* × *japonica* hybrid remains largely elusive.

Complete elucidation of the heterosis mechanism requires an investigation of the genetic, molecular, and physiological aspects. RNA sequencing (RNA-Seq) has been widely used to investigate the heterosis of rice. For example, significantly higher expressions of many genes have been detected in the superhybrid rice Liang-You-Pei-Jiu than those of parents in seven tissues [16]. A much larger number and a more diverse form of transcripts have been identified in the hybrid of Shanyou 63 than those of parents [17]. A lot of DEGs have been identified between the superhybrid Wufengyou T025 and its parents in the developing panicle, which are involved in the carotenoid biosynthesis and plant hormone signal transduction [18]. Meanwhile, many DEGs have been detected between the superhybrid XY9308 and its parents, which are closely related to root heterosis at the tillering and heading stages [3]. However, few investigations have been conducted to reveal the underpinning heterosis of salt tolerance in the inter-subspecific *indica* × *japonica* hybrid. 

Roots play significant roles in salinity stress response and tolerance in rice [19,20]. In this study, RNA-Seq was used to investigate the global transcriptomes of roots from the *indica* × *japonica* hybrid and its parents under salt stress. The DEGs and their expression patterns were analyzed. Hybridization can activate or inhibit the expression of certain genes in hybrids, which might play important roles in the heterosis of salt tolerance. Thus, the DEGs in the hybrid with the expression lower or higher than those of both parents were focused on the investigation in this study. The identified genes provide valuable clues to elucidate the heterosis of salt tolerance in rice.

## 2. Results

### 2.1. Heterosis of Salt Tolerance in Inter-Subspecific Hybrid Rice

**Figure 1:**
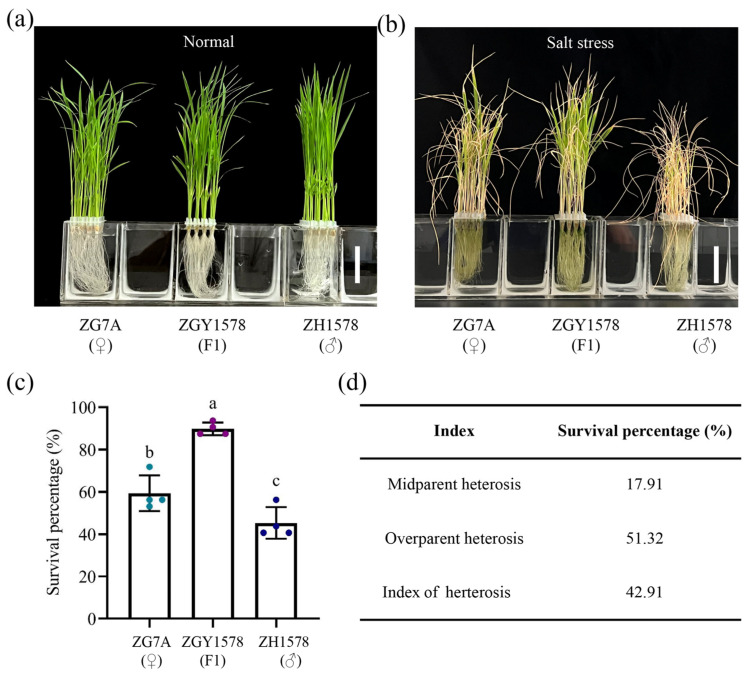
Phenotype of salt tolerance among hybrid ZGY1578 and its parents ZG7A and ZH1578. (**a**) The seedlings’ growth under normal (H_2_O) conditions for 21 days. (**b**) The seedlings’ growth after 120 mM NaCl for 3 days and with the recovery growth under normal (H_2_O) conditions for 10 days. Bars = 5 cm. (**c**) Comparison of seedling survival percentages after salt treatment. Data are means (±SD), *n* = 32. The different letters indicate the significant differences determined using ANOVA test: *p* < 0.05. (**d**) Evaluation of the heterosis of salt tolerance.

In order to identify the heterosis of salt tolerance, the seedlings after 21 days’ growth (Figure 1a) of the *indica* × *japonica* hybrid Zhegengyou1578 (ZGY1578), maternal line Zhegeng7A (ZG7A), and paternal line Zhehui1578 (ZH1578) were treated with 120 mM NaCl for 3 days, and then the seedlings were cultivated under normal (H_2_O) conditions for 10 days. After that, the survival percentage of seedling was measured. We observed that the seedling growth of ZGY1578 was more vigorous than those of either parent (Figure 1b). The survival percentage of seedling was 59.4%, 89.8%, and 45.3% in ZG7A, ZGY1578, and ZH1578 after salt treatment, respectively (Figure 1c). The values of midparent heterosis (MPH), overparent heterosis (OPH), and index of heterosis (IH) were calculated to measure the heterosis of salt tolerance. The significant MPH, OPH, and IH were observed in the survival percentage of seedling after salt treatment (Figure 1d). These results suggest that the *indica* × *japonica* hybrid rice ZGY1578 shows a superior performance of salt tolerance compared with its parents.

### 2.2. Differentially Expressed Genes among Hybrid and Its Parents

**Figure 2:**
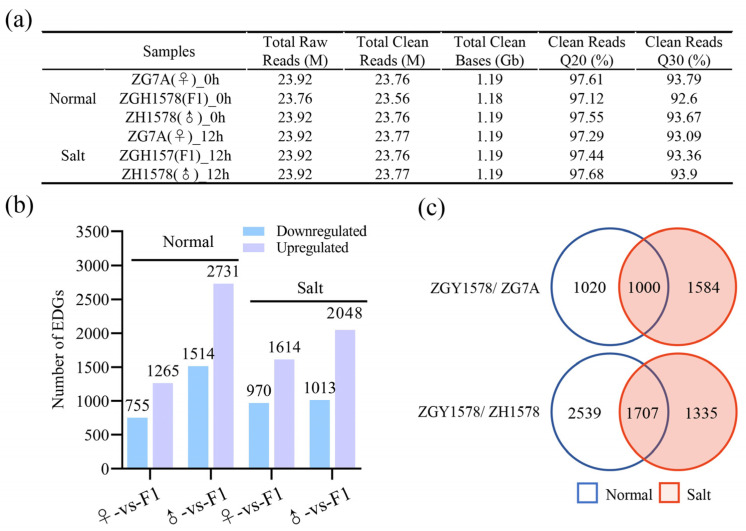
DEGs between hybrid ZGY1578 and its parents ZG7A and ZH1578. The roots of 21 days’ seedling under normal (H_2_O) and salt stress (120 mM NaCl for 12 h) conditions were harvested for RNA-Seq. (**a**) Summary data of RNA-Seq. (**b**) Up- and downregulated DEGs with at least twofold change. (**c**) Comparison of DEGs between normal and salt stress conditions.

Roots play significant roles in salinity stress response and tolerance. In order to reveal the candidate genes involving the heterosis of salt tolerance, the transcriptome changes in roots of ZGY1578 and its parents ZH1578 and ZG7A were conducted under normal (H_2_O) and salt stress (120 mM NaCl for 12 h) conditions using the RNA-Seq approach. After removing the adapters, low-quality data, and ambiguous reads, we obtained approximately 23.7 million clean reads in each sample (Figure 2a). The Phred quality score (Q20 and Q30), which was used to measure the quality of the base recognition in the sequencing process in each sample, was greater than 92%. A total of 2020 and 4245 DEGs were identified between ZGY1578 and ZG7A and between ZGY1578 and ZH1578 under normal condition, respectively, and 2584 and 3061 DEGs were identified after salt treatment (Figure 2b; Supplementary Table S1). By comparison, 1000 and 1707 DEGs were simultaneously identified under both normal and salt stress condition between ZGY1578 and ZG7A and between ZGY1578 and ZH1578, respectively, while 1584 and 1335 DEGs were specifically detected under salt stress (Figure 2c).

### 2.3. Expression Pattern of Differentially Expressed Genes

**Figure 3:**
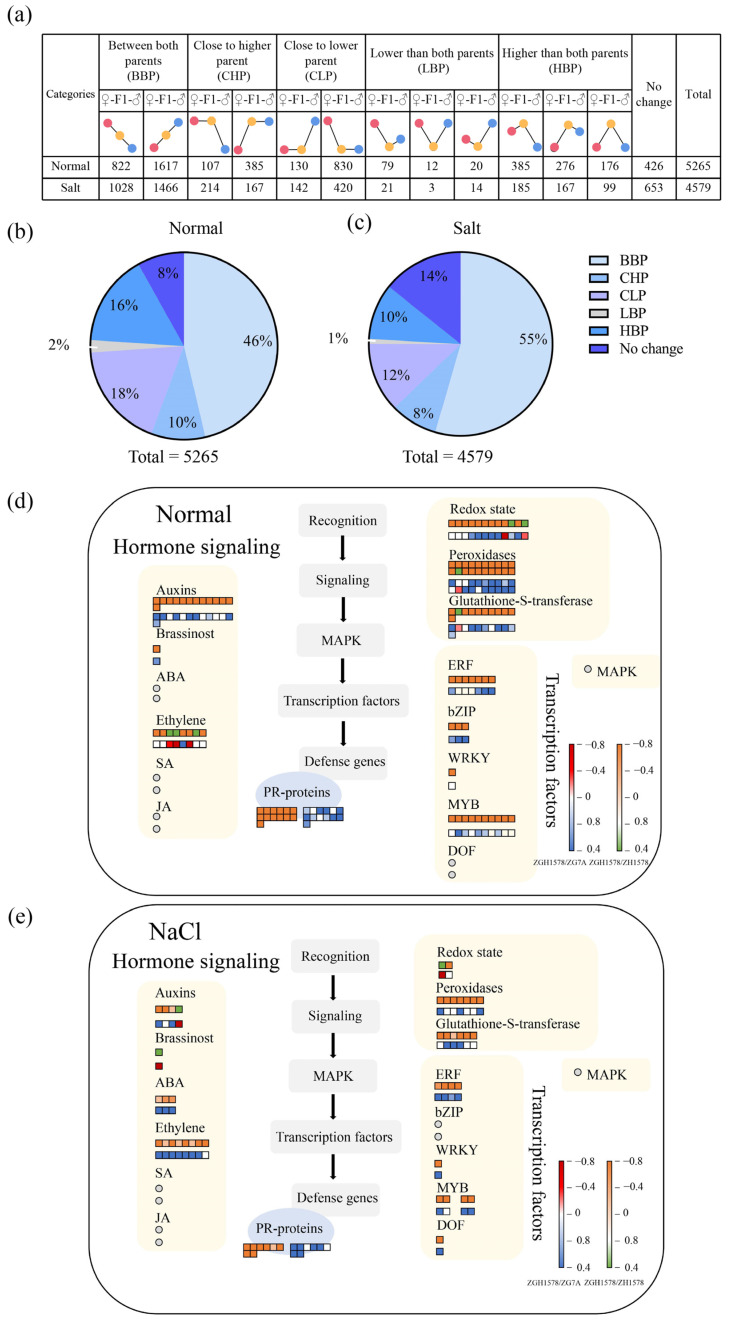
Expression pattern of DEGs under normal (H_2_O) and salt stress (120 mM NaCl for 12 h) conditions. (**a**) Classification of DEGs. The position of the color circle indicates the relative expression level. The red, yellow, and blue circles indicate the maternal parent (♀), hybrid (F1), and paternal parent (♂), respectively. The ratio of each group under (**b**) normal and (**c**) salt stress conditions. Functional classification of DEGs classified into LBP and HBP using MapMan analysis under (**d**) normal and (**e**) salt stress conditions. Values represent the log2 fold changes of the gene.

According to the gene expression levels in the hybrid relative to its parents, the DEGs were classified into five groups, including between both parents (BBP), close to the higher parent (CHP), close to the lower parent (CLP), lower than both parents (LBP), and higher than both parents (HBP) (Figure 3a). We observed that 46%, 10%, 18%, 2%, and 16% of DEGs were classified into BBP, CHP, CLP, LBP, and HBP under normal conditions (Figure 3b), respectively, and 55%, 8%, 12%, 1%, and 10% of DEGs were classified into BBP, CHP, CLP, LBP, and HBP under salt stress (Figure 3c). Hybridization can activate or inhibit the expression of certain genes in hybrids, which might provide valuable clues to elucidate the formation of heterosis. Thus, the DEGs belonging to LBP and HBP were used for further MapMan analysis. It showed that these DEGs were mainly associated with the transcription factors, hormone-, and ROS-related genes under both normal and salt stress conditions (Figure 3d,e; Supplementary Table S2). These DEGs associated with the transcription factors, hormone-, and ROS-related genes were focused on the following investigation.

### 2.4. Transcription Factors Associated with Heterosis of Salt Tolerance

**Figure 4:**
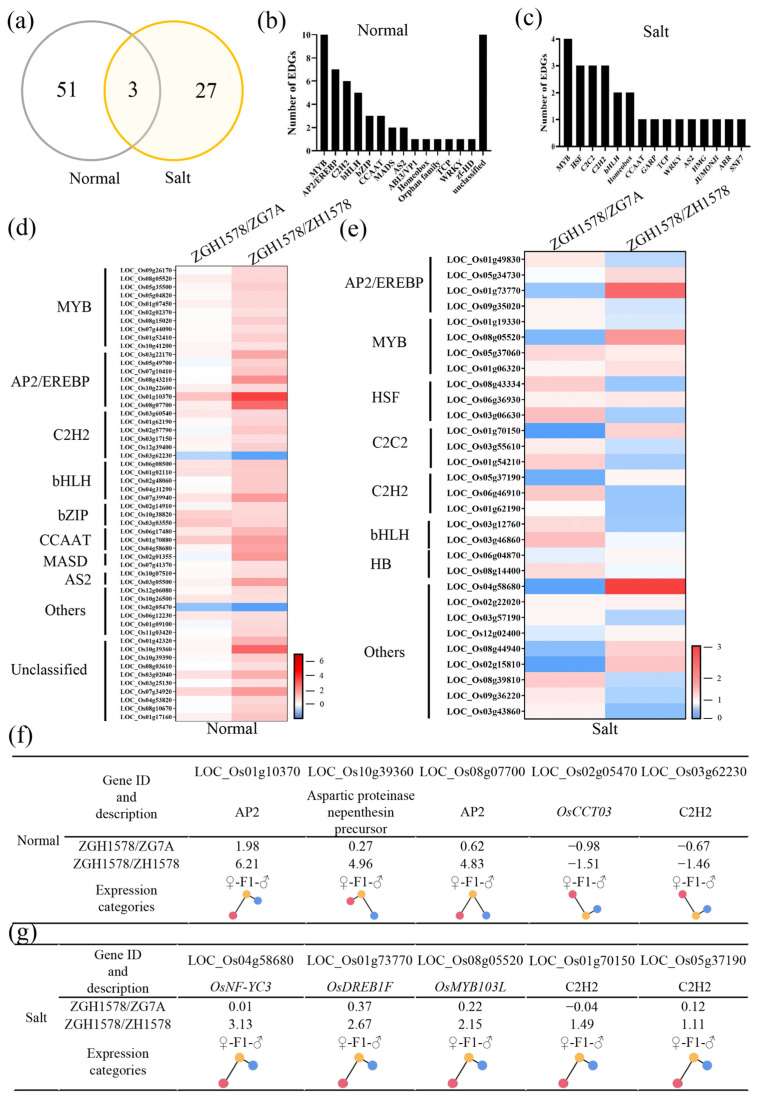
Expression pattern of DEGs associated with transcription factors under normal (H_2_O) and salt stress (120 mM NaCl for 12 h) conditions. (**a**) Comparison of DEGs between normal and salt stress conditions. Classification of DEGs under (**b**) normal and (**c**) salt stress conditions. Expression pattern of DEGs between ZGY1578 and its parents ZG7A and ZH1578 under (**d**) normal and (**e**) salt stress conditions. The details of the top 5 changed DEGs under (**f**) normal and (**g**) salt stress conditions. Values represent the log2 fold changes of the gene. The position of the color circle indicates the relative expression level. The red, yellow, and blue circles indicate the maternal parent (♀), hybrid (F1), and paternal parent (♂), respectively.

As mentioned above, a total of 54 and 30 DEGs associated with transcription factors were identified under normal and salt stress conditions, respectively, between hybrid ZGY1578 and its parents (Figure 4a). Of them, only 3 DEGs were identified under both conditions, while 27 DEGs were specifically identified under salt stress. The top 3 DEGs belonged to the MYB, AP2/EREBP, and C2H2 transcription factors under normal conditions (Figure 4b), while the top 4 DEGs belonged to the MYB, HSF, C2C2, and C2H2 transcription factors under salt stress (Figure 4c). Generally, the higher change values of gene expression in DEGs were observed between ZGY1578 and ZGH1578 compared with those of DEGs between ZGY1578 and ZG75A under both normal and salt stress conditions (Figure 4d,e). Of them, the top 5 changed DEGs were LOC_Os01g10370, LOC_Os10g39360, LOC_Os08g07700, LOC_Os02g05470, and LOC_Os03g62230 under normal condition (Figure 4f). The top 5 changed DEGs were LOC_Os04g58680, LOC_Os01g73770, LOC_Os08g05520, LOC_Os01g70150, and LOC_Os05g37190 under salt stress (Figure 4g). By comparison, the majority of these DEGs were highly expressed in the hybrid ZGY1578 compared with its parents. The induced DEGs in hybrid ZGY1578 might play important roles in the heterosis of salt tolerance.

### 2.5. Hormone-Related Genes Associated with Heterosis of Salt Tolerance

**Figure 5:**
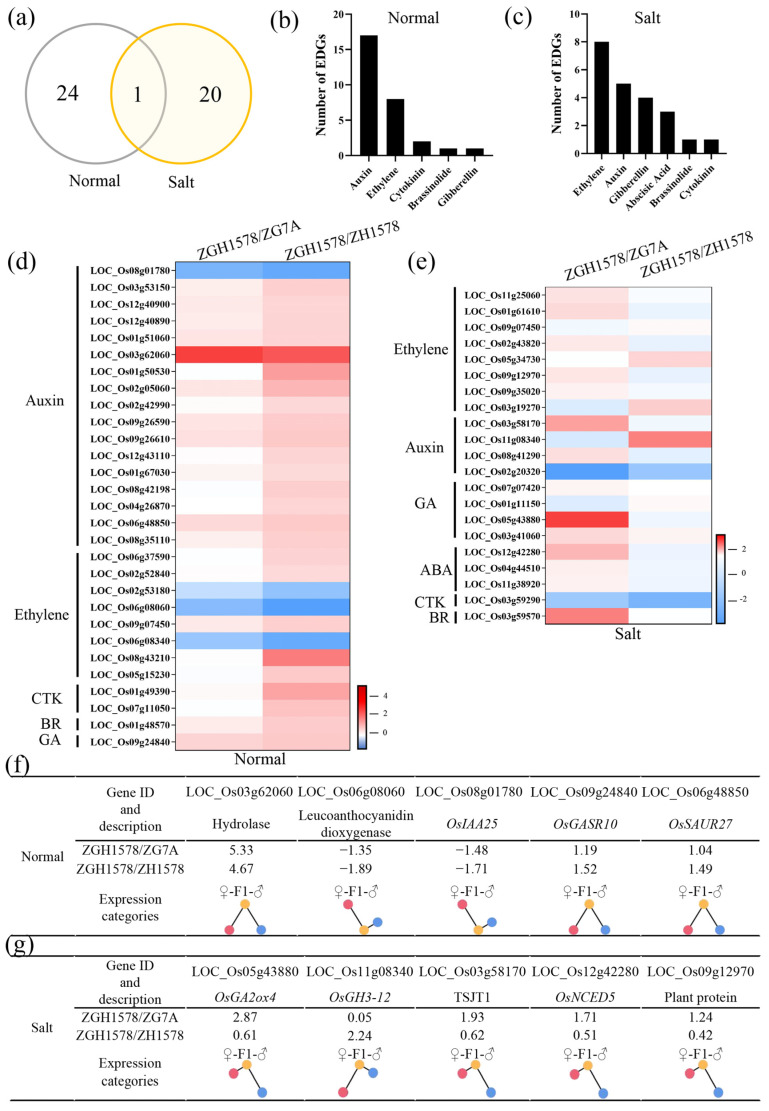
Expression pattern of DEGs associated with hormone-related genes under normal (H_2_O) and salt stress (120 mM NaCl for 12 h) conditions. (**a**) Comparison of DEGs between normal and salt stress conditions. Classification of DEGs under (**b**) normal and (**c**) salt stress conditions. Expression pattern of DEGs between hybrid ZGY1578 and its parents ZG7A and ZH1578 under (**d**) normal and (**e**) salt stress conditions. The details of the top 5 changed DEGs under (**f**) normal and (**g**) salt stress conditions. Values represent the log2 fold changes of the gene. The position of the color circle indicates the relative expression level. The red, yellow, and blue circles indicate the maternal parent (♀), hybrid (F1), and paternal parent (♂), respectively.

As mentioned above, a total of 25 and 21 DEGs associated with hormone-related genes were identified under normal and salt stress conditions, respectively, between hybrid ZGY1578 and its parents (Figure 5a). Of them, only 1 DEG was identified under both conditions, while 20 DEGs were specifically expressed under salt stress. The top 3 DEGs were auxin-, ethylene-, and cytokinin (CTK)-related genes under normal conditions (Figure 5b), while the top 3 DEGs were ethylene-, auxin-, and gibberellin-related genes under salt stress (Figure 5c). Generally, the higher change values of the gene expression in DEGs were observed between ZGY1578 and ZGH1578 compared with those of DEGs between ZGY1578 and ZG75A under normal conditions (Figure 5d). However, opposite results were observed that the higher change values of the gene expression in DEGs existed between ZGY1578 and ZG75A under salt stress (Figure 5e). Of them, the top 5 changed DEGs were LOC_Os03g62060, LOC_Os06g08060, LOC_Os08g01780, LOC_Os09g24840, and LOC_Os06g48850 under normal conditions (Figure 5f). The top 5 changed DEGs were LOC_Os05g43880, LOC_Os11g08340, LOC_Os03g58170, LOC_Os12g42280, and LOC_Os09g12970 under salt stress (Figure 5g). By comparison, the majority of these DEGs were highly expressed in the hybrid ZGY1578 compared with its parents. The induced DEGs in hybrid ZGY1578 might play important roles in the heterosis of salt tolerance.

### 2.6. ROS-Related Genes Associated with Heterosis of Salt Tolerance

**Figure 6:**
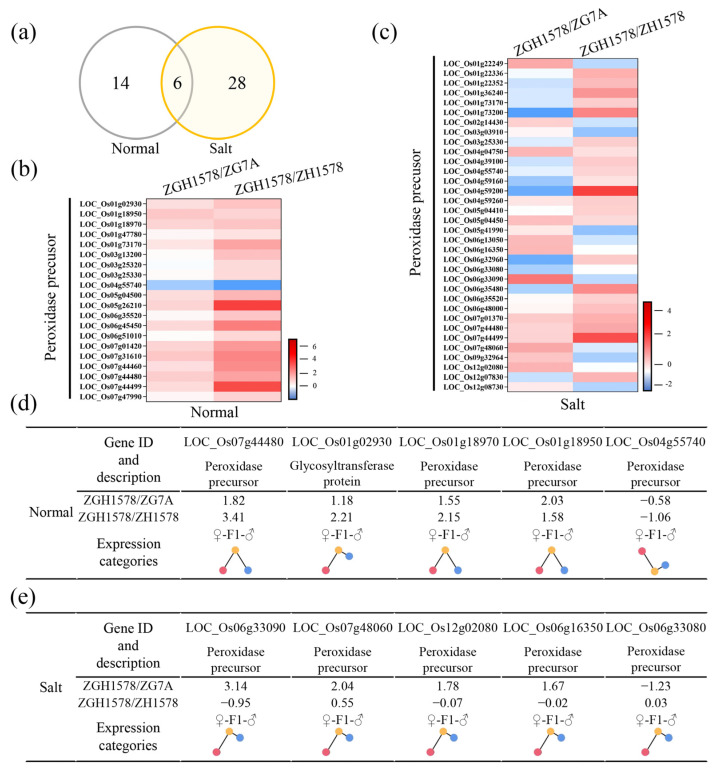
Expression pattern of DEGs associated with ROS under normal (H_2_O) and salt stress (120 mM NaCl for 12 h) conditions. (**a**) Comparison of DEGs between normal and salt stress conditions. Expression pattern of DEGs between hybrid ZGY1578 and its parents ZG7A and ZH1578 under (**b**) normal and (**c**) salt stress conditions. The details of the top 5 changed DEGs under (**d**) normal and (**e**) salt stress conditions. Values represent the log2 fold changes of the gene. The position of the color circle indicates the relative expression level. The red, yellow, and blue circles indicate the maternal parent (♀), hybrid (F1), and paternal parent (♂), respectively.

As mentioned above, a total of 20 and 34 DEGs associated with ROS-related genes were identified under normal and salt stress conditions, respectively, between hybrid ZGY1578 and its parents (Figure 6a). Of them, only 6 DEGs were identified under both normal and salt stress conditions, while 28 DEGs were specifically expressed under salt stress. Generally, higher change values of the gene expression in DEGs were observed between ZGY1578 and ZGH1578 compared with those of DEGs between ZGY1578 and ZG75A under normal conditions (Figure 6b). However, opposite results were observed that higher change values of the gene expression in DEGs existed between ZGY1578 and ZG75A under salt stress (Figure 6c). Of them, the top 5 changed DEGs were LOC_Os07g44480, LOC_Os01g02930, LOC_Os01g18970, LOC_Os01g18950, and LOC_Os04g55740 under normal conditions (Figure 6d). The top 5 changed DEGs were LOC_Os06g33090, LOC_Os07g48060, LOC_Os12g02080, LOC_Os06g16350, and LOC_Os06g33080 under salt stress (Figure 6e). By comparison, the majority of these DEGs were highly expressed in hybrid ZGY1578 compared with its parents. The induced DEGs in hybrid ZGY1578 might play important roles in the heterosis of salt tolerance.

### 2.7. Validation of DEGs by Real-Time Quantitative PCR

**Figure 7:**
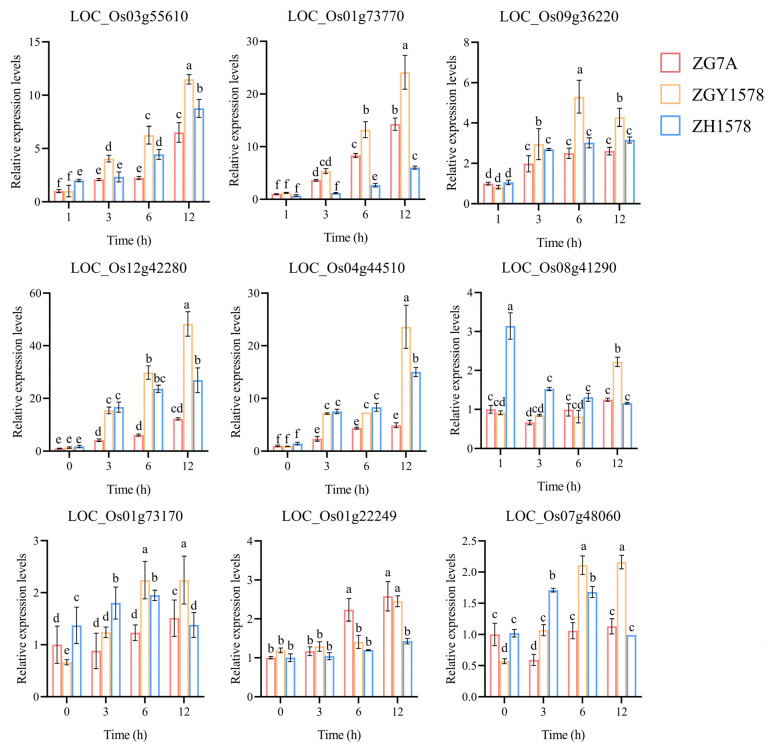
Relative expression levels of the randomly selected DEGs detected using real-time quantitative PCR. The *OsActin* gene was used as the internal control. Data are means (±SD), *n* = 3. The different letters indicate the significant differences determined using ANOVA test: *p* < 0.05.

To validate the reliability of RNA-Seq results, the expressions of several randomly selected DEGs were tested using a real-time quantitative PCR approach. The expression patterns of these genes were consistent with the results of transcriptome data (Figure 7). The expressions of transcription factor genes (LOC_Os03g55610, LOC_Os01g73770, and LOC_Os09g36220) were significantly higher in the roots of hybrid ZGY1578 compared with those of ZG75A and ZH1578 after 120 mM NaCl treatment for 6 and 12 h. Similarly, the expressions of hormone-related genes (LOC_Os12g42280, LOC_Os04g44510, and LOC_Os08g41290) and ROS-related genes (LOC_Os01g73170 and LOC_Os07g48060) were significantly higher in the roots of hybrid ZGY1578 compared with those of ZG75A and ZH1578 after 120 mM NaCl treatment for 12 h.

## 3. Discussion

Heterosis, defined as the superior performance of hybrids over their parents, is a ubiquitous phenomenon in plants [21]. In this study, we observed that the inter-subspecific hybrid ZGY1578 has significant heterosis in salt tolerance at the seedling stage in rice. It might be an elite rice cultivar for production in salinity soils. Using a strong heterosis of inter-subspecific hybrids might be the main approach to increase rice’s salt tolerance in the future. Previously, it has been revealed that differential epigenetic modifications regulate the changes of transcript levels among rice hybrids and parental lines [22]. DNA methylation is a heritable epigenetic mark that controls gene expression, which may play important roles in heterosis [23]. Similarly, we speculated that the changes of gene expression might play important roles in the heterosis of salt tolerance in rice. Genome-wide analysis of gene expression is a useful approach to detect the genes involving heterosis. Here, we attempted to reveal the genes involving the heterosis of salt tolerance, and then the gene expression was conducted in roots of inter-subspecific hybrid rice under salt stress using the RNA-Seq approach. Our data revealed a comprehensive overview of transcriptional trends associated with the heterosis of salt tolerance and provided a useful resource for the rice community.

It has been reported that the additive expression balance of genes in hybrids establishes an important foundation for heterosis [24]. Previously, the expression levels of most genes in hybrids were near the midparent value in rice [25,26]. Similarly, we observed that the expression levels of majority of DEGs in hybrids were between the parent’s values under salt stress. Hybridization can activate or inhibit the expression of certain genes in hybrids, which might play important roles in heterosis. Thus, the DEGs in hybrids with an expression value lower or higher than those of both parents were the focus of the investigation in this study. The dominance, overdominance, and epistasis might be considered the explanations for the reason of DEGs induced or inhibited in hybrids, but it needs to be further investigated. We observed that the expressions of transcription factors were significantly induced or inhibited in hybrids. For example, *OsNF-YC3* (LOC_Os04g58680), *OsDREB1F* (LOC_Os01g73770), and *OsMYB103L* (LOC_Os08g05520) were the top 3 highly expressed genes in hybrid ZGY1578 against its paternal line ZH1578. We speculate that the transcription factors might play significant roles in the heterosis of salt tolerance. Rice *OsDREB1F* has been indicated to be involved in salt, drought, and low-temperature tolerance [27,28]. Moreover, the nuclear factor Y subunit C (NF-YC) genes have been reported to be involved in the flowering and hypocotyl elongation in *Arabidopsis* [29,30,31]. *OsMYB103L* has been revealed to be involved in the secondary wall biosynthesis, leaf rolling, and mechanical strength in rice [32,33]. However, the functions of *OsNF-YC3* and *MYB103L* on salt tolerance have not been reported. The exact mechanisms of these identified genes in salt tolerance heterosis need to be further investigated.

It is well known that hormones, such as abscisic acid (ABA), auxin, and cytokinin, are involved in the root development and the adaption to environmental stresses [20,34,35]. However, whether the hormone-related genes are involved in the heterosis of salt tolerance has not been reported in rice. In this study, we observed that the expressions of several DEGs associated with hormone-related genes were significantly induced or inhibited in roots of hybrids compared with those of parents. We speculated that auxin and GA-related genes might be associated with the heterosis of salt tolerance in rice. For example, *OsGA2ox4* (LOC_Os05g43880) and *OsGH3-12* (LOC_Os11g08340) were the top highly expressed genes in hybrid ZGY1578 compared with its parents. Previously, the auxin-inactivation-related *OsCH3* genes have been revealed to be involved in the root development of rice [36,37]. Rice *OsGH3.2* has been reported to be involved in the regulation of auxin level and root architecture [37]. Meanwhile, the GA biosynthesis gene *OsGA2ox4* has been reported to be involved in the plant growth and development in rice [38,39]. It is interesting to investigate whether *OsCH3-12* and *OsGA2ox4* regulate the heterosis of salt tolerance by influencing auxin and GA levels. Moreover, several DEGs associated with peroxidases were detected in hybrid ZGY1578 compared with its parents in this study. Previously, the roles of peroxidase genes in salinity tolerance have been demonstrated in soybean and wheat [40,41]. Overexpression of the wheat peroxidase gene *TaPRX-2A* enhances the tolerance to salt stress by reducing the reactive oxygen species accumulation and malondialdehyde content [41]. We assumed here that the identified peroxidase genes might play important roles in salt tolerance in rice. How the peroxidase genes regulate the heterosis of salt tolerance needs to be further investigated in rice.

In summary, we investigated the global transcriptome of roots in inter-subspecific hybrid rice and its parents under salt stress. Several DEGs associated with transcription factors, hormones, and ROS-related genes were identified in hybrid rice, which might be involved in the heterosis of salt tolerance. Previously, transcription factors, such as *WRKY114* and *OsbZIP47*, are involved in the regulation of *OsGA2ox4* [38,39]. Meanwhile, *OsDREB2A* has the maintenance function of cellular redox homeostasis in rice [42], and the zinc finger protein regulates the peroxidase biosynthesis involving salt tolerance [43]. It interesting to reveal whether the identified transcription factors here regulate the heterosis of salt tolerance by directly regulating hormones and ROS-related genes in rice. The identified DEGs will provide valuable information to reveal the mechanisms of salt tolerance heterosis in rice.

## 4. Materials and Methods

### 4.1. Plant Materials

The *indica* × *japonica* hybrid ZGY1578 is commonly planted in Zhejiang Province of China. Here, ZGY1578, maternal line ZG7A, and paternal line ZH1578 were used. Seeds were provided by the Zhejiang Academy of Agricultural Science (Hangzhou, Zhejiang, China).

### 4.2. Evaluation of Salt Tolerance

A total of 32 seeds per genotype were cultured in one plot for 21 days under normal (H_2_O) conditions. Then, the seedlings were treated with 120 mM NaCl solution for 3 days and were recovery-cultured under normal (H_2_O) conditions for 10 days. After that, the survival percentage of seedling was measured. Three biological replications were conducted. Midparent heterosis (MPH), overparent heterosis (OPH), and index of heterosis (IH) were determined by the following formula: MPH = (F1−MP)/MP × 100%, OPH = (F1−HP)/HP × 100%, and IH = F1/MP × 100%, where MP is the average performance of two parents, F1 is the performance of the first generation (hybrid), and HP is the performance of the best parent.

### 4.3. RNA-Seq

The 21-day seedlings of the ZGY1578 hybrid and its parents ZG7A and ZH1578 were treated with 120 mM NaCl solution for 12 h, and then the roots were harvested for RNA extraction. The roots of untreated seedlings were used as the control. The total RNA was extracted using the TRIzol kit, according to the manual instruction (TransGen, www.transgen.com; accessed on 1 June 2022). The construction of the complementary DNA (cDNA) libraries and BGISEQ-500RS sequencing was performed at BGI Shenzhen Co., Ltd., Shenzhen, China. Three biological replications were conducted.

### 4.4. Differentially Expressed Gene Analysis

The clean reads were mapped onto the Nipponbare reference genome (MSU Rice Genome Annotation Project Release 7) according to Kim et al. [44]. The levels of gene expression were quantified in terms of fragments per kilo base of exon per million (FPKM) according to Li and Dewey [45]. The log2 fold changes of the gene FPKM were calculated among samples. The DEGs with at least a twofold changes were selected for further analyses.

### 4.5. Real-Time Quantitative RT-PCR Analysis

Total RNA was extracted using the Plant RNA Kit (Solarbio, Shanghai, China) and the first-strand cDNA was synthesized using the Biosharp Reverse Transcription Kit (Labgic, Beijing, China), according to the manufacturer’s instructions. Quantitative RT-PCR was performed using the CFX96 Real-Time System (Bio-Rad, Hercules, CA, USA), according to Zhao et al. [46]. The PCR conditions were as follows: 95 °C for 2 min, followed by 40 cycles of 95 °C for 5 s and 60 °C for 10 s. The rice *OsActin* gene was used as an internal control, and the comparative method was used to normalize transcript levels [47]. Primers are listed in Supplementary Table S3. Three biological replications were conducted.

### 4.6. Data Analysis

Experimental data were analyzed using the SAS software (Cary, NC, USA). Significant differences among samples were compared using analysis of variance (ANOVA).

## Data Availability

Not applicable.

## Supplementary Materials

The following supporting information can be downloaded at: https://www.mdpi.com/article/10.3390/ijms24032212/s1.
